# A novel biomarker of MMP-cleaved cartilage intermediate layer protein-1 is elevated in patients with rheumatoid arthritis, ankylosing spondylitis and osteoarthritis

**DOI:** 10.1038/s41598-023-48787-x

**Published:** 2023-12-07

**Authors:** Helena Port, Cecilie Møller Hausgaard, Yi He, Walter P. Maksymowych, Stephanie Wichuk, Dovile Sinkeviciute, Anne-Christine Bay-Jensen, Signe Holm Nielsen

**Affiliations:** 1 Department of Clinical Medicine, University of Copenhagen, Copenhagen, Denmark; 2 Immunoscience, Nordic Bioscience, Herlev Hovedgade 205–207, 2730 Herlev, Denmark; 3 Department of Medicine, University of Alberta, Edmonton, Alberta Canada

**Keywords:** Diagnostic markers, Predictive markers, Osteoarthritis, Rheumatoid arthritis, Ankylosing spondylitis, Cartilage

## Abstract

Rheumatic joints have an altered cartilage turnover. Cartilage intermediate layer protein 1 (CILP-1) is secreted from articular chondrocytes and deposited into the cartilage extracellular matrix. We developed an immunoassay targeting a Matrix Metalloproteinase (MMP)-generated neo-epitope of CILP-1, named CILP-M. Human articular cartilage was cleaved with proteolytic enzymes and CILP-M levels were measured. We also quantified CILP-M in two studies from patients with rheumatoid arthritis (RA), ankylosing spondylitis (AS) and osteoarthritis (OA) and explored the monitoring and prognostic potential of CILP-M in TNF-α inhibitory treatment and modified Stoke AS Spine Score (mSASSS) progression. CILP-M was generated by MMP-1, -8 and -12. In the discovery study, CILP-M was significantly higher in patients with RA, AS and OA than healthy donors (p < 0.01, p < 0.001, p < 0.05) with an area under the curve (AUC) between the diseased groups and healthy donors > 0.95 (p < 0.001). In the validation study, patients with RA and AS had significantly higher CILP-M levels than healthy controls (p < 0.001) and AUC > 0.90 (p < 0.001). Patients with AS treated with TNF- α inhibitory treatment in the validation study had significantly lower CILP-M levels after treatment (p = 0.004). CILP-M may provide useful insights into cartilage degradation processes in rheumatic diseases.

## Introduction

Articular cartilage is a heterogenous tissue where cells are organized into a matrix. The matrix is formed by chondrocytes and its major constituent are fibril forming collagens, primarily, type II and IX collagen, and large aggregating proteoglycans^1^. To form the cartilage matrix, other non-collagenous extracellular matrix proteins, such as the cartilage intermediate layer protein (CILP) are also present ^2^. CILP-1 is a glycoprotein, first identified in articular cartilage, and believed to have a role in cartilage scaffolding^2^. It is secreted from articular chondrocytes^2^, which are responsible for maintaining cartilage homeostasis by low-turnover remodelling of matrix proteins^3^. A hallmark of rheumatic joint diseases is altered cartilage turnover, leading to dysregulated composition. After CILP-1 is secreted from articular chondrocytes, it is deposited into the cartilage extracellular matrix ^2^ and cleaved by proteases expressed during joint inflammation, generating neo-epitope fragments, which are released into the circulation ^2, 4^. It has been shown that synthesis of CILP is increased in early osteoarthritis (OA) cartilage^5^ and is associated with other musculoskeletal disorders ^6, 7^.

CILP inhibits tumour growth factor (TGF)β, preventing ECM cell proliferation and anabolism promoted by TGF in intervertebral disc disease^8^. In previous studies, CILP-1 was found to be highly expressed in intervertebral discs and its expression increased in lumbar disc disease^9–11^. Additionally, CILP functions as an antagonist of Insulin-like growth factor 1 (IGF1) by inhibiting ligand-induced IGF1 receptor. IGF1 is a critical factor involved in promoting chondrocyte anabolism and proliferation^8^. An immune response to CILP has also been suggested to contribute to the pathogenesis of inflammatory joint destruction present in rheumatoid arthritis (RA) and OA^12^. Moreover, *CILP* mRNA expression was observed in synovial tissue from patients with OA^13^, indicating its expression is not restricted to articular cartilage. These findings suggest that CILP proteins play a role in cartilage structure and reflect the extent of inflammation and tissue turnover in rheumatic diseases. Nevertheless, no data is currently available regarding the role of cleaved CILP-1 and its potential as a marker for disease activity, as exemplified in the monitoring of anti- inflammatory treatments such as anti-TNF-α, and for prognosis in terms of structural damage progression. Moreover, studies investigating the role of CILP-1 in other joint-related diseases such as ankylosing spondylitis (AS) are needed to further elucidate its function and implication in cartilage structure and pathology. Biomarkers reflecting disease activity and prognosis of rheumatic diseases such as RA, AS and OA are a high priority unmet clinical need to monitor patients on treatment for progression of these diseases.

In this present work we developed an immunoassay targeting an MMP-1, MMP-8, and MMP-12-generated neo-epitope of CILP-1, named CILP-M. The clinical value of CILP-M was evaluated in joint-related diseases (RA, AS and OA) in two exploratory patient studies. We also explored the disease activity monitoring and prognostic potential of CILP-M in AS patients receiving anti-TNF-α therapy using clinical measures of disease activity and the modified Stoke AS Spine Score (mSASSS) progression score.

## Results

### Technical evaluation and characterization of CILP-M

**Figure 1:**
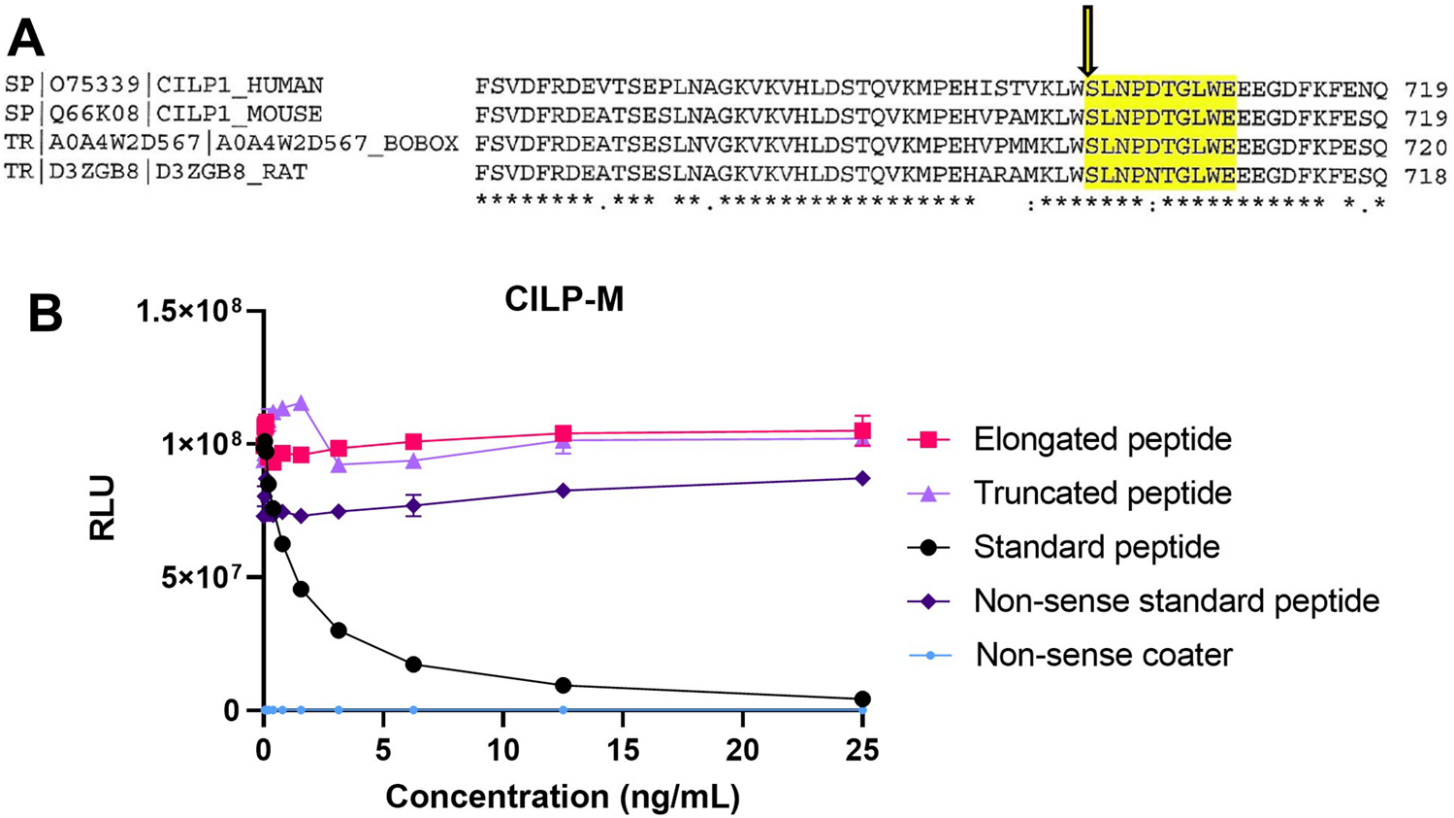
Alignment and specificity of the CILP-M assay. (**A**) Sequence alignment of the targeted sequence for CILP-M in human with mouse, bovine, and rat. Sequence is highlighted in yellow, and protease cleavage is marked with an arrow. (**B**) Specificity of the CILP-M assay. Reactivity towards the standard peptide (SLNPDTGLWE), truncated peptide (NPDTGLWE), elongated peptide (WSLNPDTGLWE) and non-sense standard peptide and coater (DSGPEYADVV). Signals are shown as relative luminescence (RLU) per second, as a function of standard peptide. Abbreviations: LLOQ, lower limit of quantification; ULOQ, upper limit of quantification.

The monoclonal antibody (mAb) clone NB326#76 13B5-1C6-2C4 showed the best native reactivity, peptide affinity, and stability for the assay and was chosen for assay development. A summary of the technical evaluation of the CILP-M assay can be found in Table 1. Briefly, the measurement range (lower limit of quantification (LLOQ)- upper limit of quantification (ULOQ) was determined to be 0.54–30.00 ng/mL. The mean inter- and intra-assay variation was 10.3% and 7.2% respectively, and linearity was approved from undiluted to a two-fold dilution in human serum. The stability of the analyte was acceptable both during prolonged storage (2 h, 4 h, 24 h and 48 h) of human serum samples at 4 °C and 20 °C (95% and 92%, respectively), and three freeze–thaw cycles (92–119%). Haemoglobin, lipemia and biotin did not interfere with measurements of CILP-M in human serum. The human sequence was aligned using UNIPROT, and the corresponding sequence in mouse and bovine is 100% aligned, while rat has one mismatch in position 5 (Fig. 1A). To evaluate the specificity of the CILP-M assay, the mAb was tested towards the elongated peptide, truncated peptide, non-sense standard peptide and non-sense coater, and showed no reactivity towards those peptides (Fig. 1B).

**Table 1:** Summary of technical parameters for CILP-M.

Technical validation	Results
IC50	2.0 ng/mL
Measurement range (LLOQ-ULOQ)	0.54–30 ng/mL
LLOB	0.40 ng/mL
Inter-assay variation, mean (range)	10.3% (6.52–15.25)
Intra-assay variation, mean (range)	7.2% (4.13–10.49)
Dilution recovery of human normal serum	112.4% (106.0–116.0)
Spiking recovery (serum in selection peptide)	98% (88–103)
Mean analyte recovery 48 h, 4 °C/20 °C	95% (72.2–110.2)/92% (76.4–123.1)
Mean analyte recovery, 3 freeze/thaw cycles	100.8% (92–119)
Haemoglobin interference, mean recovery (low/high)	75% (64–84.8)/85% (84.2–85.2)
Lipemia interference, mean recovery (low/high)	97% (92.7–101.7)/97% (92.1–100.9)
Biotin interference	> 100 ng/mL

**Figure 2:**
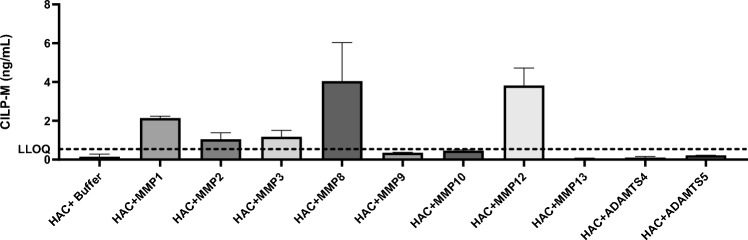
In vitro cleavage of human articular cartilage by ECM remodelling enzymes. The cleaved human articular cartilage by MMP1, MMP2, MMP3, MMP8, MMP9, MMP10, MMP12, MMP13, ADAMTS4 and ADAMTS5 was measured by the CILP-M competitive chemiluminescence immunoassay (CLIA) assay, data is shown as mean + SD.

To investigate the responsible enzyme for CILP-M generation, human articular cartilage was cleaved with a panel of enzymes. Based on measurements of CILP-M ELISA assay, CILP-M was primarily generated by MMP-1, MMP-8 (with the highest concentration) and MMP-12 (Fig. 2). These results were further confirmed by Western blot analysis, where CILP-M mAb detected bands at ~ 54 kDa when HAC was cleaved with MMP-1, MMP-3, MMP-8 (the most prominent), and MMP-12, and less prominent bands when cleaved with MMP-3 and MMP-9 (Supplementary Fig. S1). The neo-epitope of CILP-M starts at position 699 of CILP-1 protein, with a length of 484 amino acids, which equals a weight of 54 kDa^14^. A band at ~ 80 kDa was also detected when HAC was cleaved with MMP-1, MMP-2, MMP-3, MMP-8, MMP-9, and MMP-12. It could be that the fragment of interested was attached to glycans or other sugars due to post-translational modifications^14^.

### Baseline demographic and clinical characteristics

Tables 2 and 3 show the demographic characteristics of the two independent used cohorts. In the discovery cohort, a significant difference was found between the ages of the groups (p < 0.0001). The significant difference was driven by patients with OA, who on average are 30 years older than healthy donors, RA and AS in this cohort. There was no difference in sex distribution between the groups. In the validation cohort, there was also a significant difference between the age of the groups (p < 0.0001), with RA patients being on average 15 years older than the healthy donors and AS patients. In addition, there were more men in the RA and AS groups compared to the healthy donors (p < 0.0001).

**Table 2:** Patient demographics for the discovery cohort.

	Healthy (N = 13)	Rheumatoid arthritis (N = 18)	Ankylosing spondylitis (N = 14)	Osteoarthritis (N = 8)	p-value
Age (years)	45.02 (8.97)	35.72 (3.30)	35.71 (3.10)	69.88 (3.31)	< 0.001
Sex, male	6 (46.2%)	9 (50.0%)	8 (57.1%)	4 (50.0%)	0.952
BMI	–	24.11 (1.80)	22.63 (1.70)	27.64 (3.66)	0.594
Ethnicity					
Unknown	–	8	8	–	
Caucasian	12 (92.3%)	10 (100.0%)	6 (100.0%)	8 (100.0%)	
Hispanic	1 (7.7%)	0 (0.0%)	0 (0.0%)	0 (0.0%)	
CILP-M (ng/mL)	0.76 (0.22)	1.85 (0.75)	1.97 (0.99)	1.92 (0.85)	< 0.001

Categorical variables are written as number (percentage), while continuous variables are mean (standard deviation). Kruskal–Wallis rank test was used to compare differences among the groups.

*BMI* body mass index.

**Table 3:** Patient demographics for the validation cohort.

	Healthy (N = 105)	Rheumatoid arthritis (N = 23)	Ankylosing spondylitis (N = 146)	p-value
Age (years)	41.23 (12.69)	57.78 (10.94)	44.36 (12.67)	< 0.001
Sex (male)	53 (52.0%)	18 (78.3%)	113 (77.4%)	< 0.001
Disease duration (years)	–	13.45 (11.26)	20.92 (13.72)	
Anti-TNF-α				
Etanercept	–	14 (60.9%)	27 (85.6%)	
Adalimumab	–	–	18 (12.3%)	
Infliximab	–	9 (39.1%)	44 (30.1%)	
Golimumab	–	–	8 (5.5%)	
No (NSAIDs)	–	–	49 (33.56%)	
Baseline CRP (mg/L)	–	26.93 (38.86)	14.69 (20.24)	
Baseline ESR (mm/h)	–	34.73 (27.00)	21.50 (19.19)	
Baseline BASDAI	–	–	5.58 (2.36)	
Baseline mSASSS	–	–	17.09 (19.60)	
2 years follow-up mSASS			19.84 (21.13)	
Patients with change (> 0) in mSASSS at 2 years follow-up			100(68%)	
CILP-M (ng/mL)	1.06 (0.57)	2.78 (1.87)	2.45 (1.03)	< 0.001

Categorical variables are written as number (percentage), while continuous variables are mean (standard deviation). Kruskal–Wallis rank test was used to compare differences among the groups.

*CRP* C-reactive protein, *ESR* erythrocyte sedimentation rate, *BASDAI* Bath Ankylosing Spondylitis Disease Activity Index, *mSASSS* modified Stoke Ankylosing Spondylitis Spinal Score.

### CILP-M is upregulated in rheumatic diseases in the discovery and validation cohort

**Figure 3:**
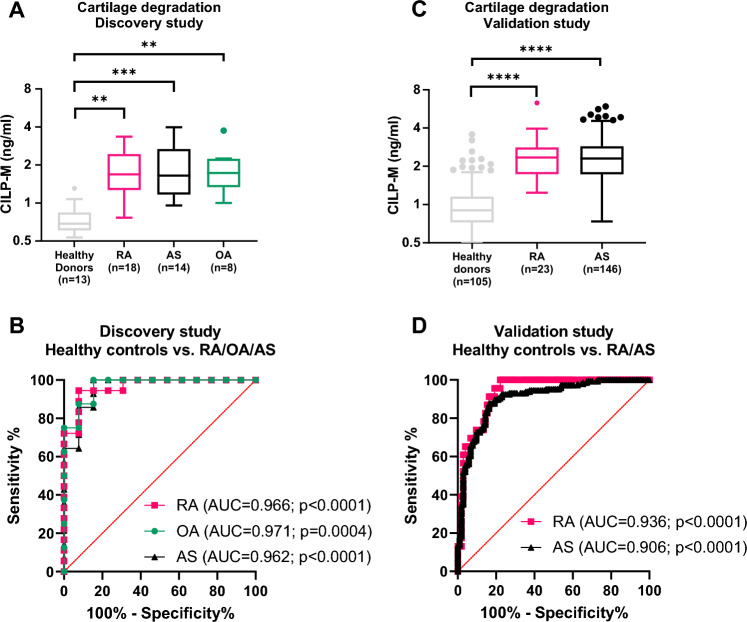
Levels of CILP-M in the discovery and validation cohort, together with receiver operating characteristic (ROC) curves. (**A**) CILP-M levels in the discovery cohort; (**B**) ROC curve analysis evaluating the ability of CILP-M to discriminate between healthy controls and RA//OA/AS respectively, in the discovery cohort. Data is shown as Tuckey plots; (**C**) CILP-M levels in the validation cohort; (**D**) ROC curve analysis evaluating the ability of CILP-M to discriminate between healthy controls and RA/AS respectively, in the validation cohort. Data was analysed using ANCOVA test (adjusting for age and gender in the validation cohort) and Area under the receiver operating characteristic curve (AUROC) analysis. Abbreviations: RA, rheumatoid arthritis; AS, ankylosing spondylitis; OA, osteoarthritis; AUC, Area Under the Curve.

In the discovery cohort, patients with RA, AS and OA showed significantly higher levels of CILP-M compared to healthy donors (p = 0.001 p = 0.0007, and p = 0.006, respectively, Fig. 3A). No difference was found between the patient groups. The diagnostic power (Area under the receiver operating characteristic (AUROC) curve analysis) of CILP-M for patients suffering from RA compared to healthy donors was 0.97 (95% CI 0.91–1.00, sensitivity = 0.94, specificity = 0.92, p < 0.0001), OA compared to healthy donors 0.97 (95% CI 0.91–1.00, sensitivity = 1, specificity = 0.85, p = 0.0004), and AS compared to healthy donors 0.96 (95% CI 0.90–1.00, sensitivity = 1, specificity = 0.85, p < 0.0001), as shown in Fig. 3B.

In the validation cohort, patients with RA and AS confirmed higher levels of CILP-M in patients with rheumatic diseases (both p < 0.0001, Fig. 3C). The diagnostic power of CILP-M for patients suffering from RA compared to healthy donors was 0.94 (95% CI 0.89–0.98, sensitivity = 1, specificity = 0.77, p < 0.0001), and AS compared to healthy donors 0.91 (95% CI 0.87–0.95, sensitivity = 0.88, specificity = 0.83, p < 0.0001), as shown in Fig. 3D. We also observed that C-reactive protein (CRP) and erythrocyte sedimentation rate (ESR)were weakly correlated with CILP-M at baseline in patients with AS (Spearman’s ρ 0.3 and 0.11, p < 0.01 and p < 0.001, respectively) No significant correlations were observed between CILP-M and the remaining clinical scores in patients with AS or RA.

**Figure 4:**
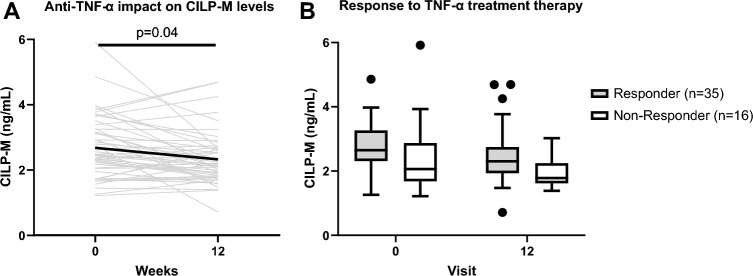
Subset analysis of CILP-M in patients with AS from the validation study. (**A)** CILP-M serum levels at baseline (week 0) and after treatment (12 weeks) in patients with AS (n = 42). Biomarker levels are presented in spaghetti plot with mean highlighted in bold. Paired t-test was performed; (**B**) CILP-M serum levels in BASDAI responders (n = 35) and non-responders (n = 16) to TNF inhibitor in patients with AS at baseline (week 0) and after 12 weeks. Data is shown as Tukey box plots. A linear mixed model was used.

Furthermore, we investigated the response to treatment based on a 50% reduction of Bath Ankylosing Spondylitis Disease Activity Index (BASDAI). We observed that after 12 weeks of treatment, levels of CILP-M were significantly decreased (p-value 0.04, Fig. 4A). However, CILP-M could not significantly discriminate between BASDAI responders and non-responders at baseline or week 12 even though responders tended to have higher levels of CILP-M compared to non-responders (p = 0.09 and p = 0.05, respectively, Fig. 4B). In addition, mSASSS progressors tended to have higher levels of CILP-M than the non-progressors (data not shown).

## Discussion

In this study, we developed and characterized a competitive ELISA for detection of CILP-M using a mAb targeting the MMP-generated neo-epitope of CILP-1. The main findings for this study were: (1) a technically robust and specific assay towards the CILP-M neo-epitope was developed; (2) CILP-M was primarily generated by MMP-1, MMP-8 and MMP-12, confirmed by the CILP-M ELISA assay and Western blot; (3) CILP-M was measurable in human serum, and the levels of CILP-M were increased in patients with RA, AS and OA as shown in the discovery and validation cohort; (4) CILP-M was also able to discriminate between patients with RA, AS or OA and healthy donors with AUC > 0.90–0.95) CILP-M levels decreased after anti-TNF-α therapy in patients with AS.

The CILP-M assay was characterised as a technically robust and accurate assay by showing acceptable dilution recovery, interference, and stability tests. The inter and intra-variation was accepted with 10.3% and 7.2%, respectively. The epitope CILP-M target was previously found by mass spectrometry of human articular cartilage^4^, which was primarily generated by ADAMTS-5. To confirm which protease generated the CILP-M cleavage site, we tested a series of proteases. In contrast with the previous finding, our in vitro cleavages showed that CILP-M epitope was generated primarily by MMP-1, MMP-8, and MMP-12 measured by the competitive chemiluminescence immunoassay (CLIA) assay and Western blot. However, the cleavage experiments differed from Zhen et al. in several aspects. In this study, we used end-stage osteoarthritic human articular cartilage, while Zhen et al. used samples derived from two separate subjects: one sample exhibiting OA pathologic features and another one with no signs of arthritis. In addition, we used a 3-day incubation period in the cleavage experiment, in contrast to the 21 days in the previous study; thus, the fragments of CILP-1 that Zhen et al. found might have been released on later days of the cleavage. In Western blot we observed the most prominent band at ~ 54 kDa when cleaved by MMP-8. Post-translational modifications (such as glycosylation) occurring close to the neo-epitope sequence could explain the larger sized immunoreactive bands observed in the Western blot^14^. Destruction and fusion of articular cartilage is a key feature in rheumatic diseases ^15–17^. Serine proteases and MMPs are highly expressed in the ECM when joint inflammation is occurring and are responsible for cartilage degradation^17^. ADAMTS4 and ADAMTS5 are considered the main aggrecan degrading enzymes in cartilage^3^, whereas MMP can degrade all components of the ECM^18^. MMP-1 and MMP-8 are two of the most common collagenases that are important for degradation of collagens in cartilage^18^. MMP-1 is produced primarily by synovial cells lining the joints and has a predominant role in OA since it controls the process of collagen degradation^19^. High levels of CILP-1 have been found in synovial tissue^13^, which could be further cleaved by MMP-1. MMP-8 is expressed at sites of inflammation and has been associated with a wide range of inflammatory disorders^17, 20^, whereas MMP-12 is secreted by inflammatory macrophages^21^, and it has been found that increased MMP-12 expression in macrophages worsened the development of inflammatory arthritis in rabbits^22^. All these findings together suggest that higher expression of MMP-1, MMP-8, and MMP-12 in inflammation sites of the joints, specifically adjacent to articular cartilage, could increase the cleavage of CILP-1, generating the neo-epitope of CILP-M.

Previous studies have investigated cleavage products of CILP-1 in rheumatic diseases, specifically in OA^23, 24^. We found that levels of CILP-M were increased in RA, AS and OA, compared to healthy donors, but did not distinguish between the different rheumatological disorders. In previous studies, CILP-1 has been associated with cartilage degenerative diseases, especially OA^6, 7^. It has also been shown that anti-CILP antibodies are present in OA and RA patients, suggesting that the autoimmune response against CILP is related to the pathogenesis of OA and RA^12^. From our current understanding, this is the first study investigating the role of CILP-1 in AS patients. Our findings are in line with previous studies indicating that patients with AS have a higher extracellular matrix remodelling in cartilage^25, 26^. However, CILP-1 has also been related to pulmonary hypertension and cardiac fibrosis, suggesting it is not specific to articular cartilage ^27–29^.

There is an unmet need for better tools to monitor disease activity and response to treatment in patients with AS. We therefore investigated if CILP-M was able to discriminate the treatment effect and response to anti-TNF-α therapy. We observed that levels of CILP-M were significantly lower after treatment, and patients responding to anti-TNF- α therapy had a trend of higher levels of CILP-M at baseline and after 12 weeks of treatment compared to non-responders. We also explored whether CILP-M was prognostic for radiographic progression, assessed by the mSASSS. Here we found patients who progressed during a 2-year period, tended to have higher levels of CILP-M but did not reach statistical significance. This is in agreement with previous findings, where cartilage degradation has been associated with radiographic progression in the spine of patients with AS^30^. However, the nature of this study was exploratory and further investigation is needed to elucidate the role of CILP-M as potential marker to monitor treatment response to anti-TNF-α therapy and disease activity.

Limitations of this study are the small population size, no information about biological variation (exercise activity, meals, time of day samples were extracted), limited clinical data available in the discovery cohort, and serum sample availability in the validation cohort. Furthermore, the healthy patients did not come from the same source as the rheumatic patients in neither the discovery nor the validation cohort. The present work was an exploratory research and cohorts with larger samples and more accurate study design might be useful for validating CILP-M in future research.

In conclusion, a novel neo-epitope biomarker CILP-M, measuring a MMP-1-, MMP-8- and MMP-12-mediated fragment of CILP-1 was developed and validated for use in human serum samples. CILP-M was upregulated in patients with RA and AS compared to healthy controls in two independent cohorts and declined levels were observed after TNF-α therapy in patients with AS. Based on these results, CILP-M may be useful to assess cartilage remodelling in joint degenerative diseases.

## Materials and methods

All reagents used were high quality chemicals from Merck (Whitehouse Station, NJ, USA) and Sigma (St. Louis MO, USA) unless stated otherwise. All synthetic peptides used for antibody production and assay validation were purchased from Genscript (Piscataway, NJ, US) (Table 4).

**Table 4:** Sequences of the synthetic peptides used for mAb production, assay development and validation.

Peptide type	Sequence
Immunogenic peptide	SLNPDTGLWE-GGC-KLH*
Selection peptide	SLNPDTGLWE
Elongated selection peptide	WSLNPDTGLWE
Truncated selection peptide	NPDTGLWE
Non-sense coating and standard peptide	DSGPEYADVV

*Keyhole Limpet Hemocyanin.

### MAb development, production, and characterization

Before the mAb generation, a cross-reactivity assessment was conducted by performing a Blast analysis (https://blast.ncbi.nlm.nih.gov and https://npsa-prabi.ibcp.fr) with the 10-amino acid (aa) sequence SLNPDTGLWE. In the analysis, we focused on the first 6 amino acids (aa), checking for the presence of a single mismatch, as well as on the entire 10-aa sequence, allowing for a maximum of two mismatches. The results showed a complete 100% match with the CILP-1 protein, with no observed mismatches. Hence, mAbs were generated using the aa sequence ↓700SLNPDTGLWEas the antigen. Four-to six-week-old balb/C mice were immunized with a subcutaneous injection of 200 μl emulsified antigen and 100 μg immunogenic peptide (SLNPDTGLWE-GGC-KLH) using Stimmune (Thermo Fisher). Immunizations were repeated every two weeks until stable serum antibody concentrations were achieved. The mouse with the highest serum concentration was selected for fusion and rested for a month before being intravenously boosted with 50 μg immunogenic peptide in 100 μl 0.9% NaCl solution. Mouse spleen cells were fused with SP2/0 myeloma cells to produce hybridoma cells as described by Gefter et al.^13^. The clones were screened for reactivity using an indirect ELISA on streptavidin-coated plates using SLNPDTGLWE-K-Biotin as the screening peptide and SLNPDTGLWE as the standard peptide to test for specificity. Hybridoma cell supernatant was purified using HiTrap affinity columns (GEHealthcare Life Science, Little Chalfront, Buckinghamshire, UK) according to the manufacturer’s instructions, and the antibody isotype was determined using a Rapid ELISA Mouse mAb Isotyping Kit (Invitrogen, Carlsbad, CA, USA). The mAb was specifically selected to recognize the standard peptide (^700^SLNPDTGLWE, 10aa), but not elongated peptide (^699^WSLNPDTGLWE, 11aa), or truncated peptide (^702^LNPDTGLWE, 9aa), ensuring the mAb is specific for the W↓S cleavage product.

The native reactivity of the antibody was assessed using human serum, citrate plasma, heparin plasma, EDTA plasma, and rat serum purchased from a commercial supplier (Valley Biomedical, Winchester, VA).

### CILP-M assay development

The development of the CLIA included several preliminary optimizing experiments where reagents, concentrations, incubation-time and -temperature were analysed by several tests. The CILP-M CLIA procedure was as follows: a 96-well streptavidin-coated white microplate (Greiner Bio-One, Kremsmünster, Austria) was coated with 2.5 ng/mL biotinylated synthetic peptide (SLNPDTGLWE-K-Biotin) dissolved in assay buffer (10 mM phosphate buffered saline (PBS), 1% bovine serum albumin, 0.1% Tween-20, 0.36% Bronidox, 4 g/L NaCl, adjusted to pH 7.4 at 20 °C) and incubated for 30 min at 20 °C with constant shaking (300 rpm) in darkness. Next, 20 μL/well of standard peptide (100 ng/mL) and samples were added to the appropriate wells, followed by the addition of 100 μL/well of HRP-labelled antibody diluted in assay buffer to the concertation of 100 ng/mL and incubated for 1 h at 20 °C with constant shaking (300 rpm) in darkness. After each incubation step, wells were washed five times with standard washing buffer (20 mM Tris, 50 mM NaCl, pH 7.2). To prepare the chemiluminescence substrate (Roche, BM Chemiluminescence ELISA substrate (POD), Basel, Switzerland), working solutions were mixed 15 min prior to use. Next, 100 μL/well of the solution was added to the plate and incubated for 3 min at 20 °C with constant shaking (300 rpm) in darkness. Relative light units were measured at all wavelengths within 5 min on a microplate luminometer reader (SpectraMax L, Molecular Devices, CA, USA). A standard curve was plotted using a 4-parameter logistic curve fit Y = (A − D)/(1 + (x/C)^B) + D, where R###0.9. Data were analysed using SoftMax Pro version 7.0.3 software.

### Technical evaluation

Two-fold dilutions of four human serum samples were used to assess linearity. Linearity was calculated as a percentage of recovery of the undiluted sample. Antibody specificity was calculated as percentage of signal inhibition by two-fold diluted standard peptide (^700^SLNPDTGLWE, 10aa), elongated peptide (^699^WSLNPDTGLWE, 11aa), truncated peptide (^702^LNPDTGLWE, 9aa), and non-sense peptide (DSGPEYADVV, 10 aa). The intra- and inter-assay variation was determined by 10 independent runs of eight quality controls and two kit controls run in double determinations. Accuracy of the assay was measured in healthy human serum samples spiked with standard peptide and a serum sample with a known high CILP-M concentration and calculated as the percentage recovery of the measured value and the expected concentration of the peptide or the serum sample with high CILP-M plus the concentration of the analyte in serum. Analytical interference was performed by adding a low/high content of haemoglobin (2.50/5 mg/mL), lipemia/lipids (1.50/5 mg/mL) and biotin (3/9 ng/mL) to a serum sample of known concentration. Recovery percentage was calculated with the normal serum sample as reference. The standard reference values for haemoglobin, lipidaemia/lipids and biotin were 0–10 mg/dL (0–0.00161 mmol/L), < 150 mg/dL (< 1.6935 mmol/L) and 0.221–3.004 ng/mL, respectively. The interference was calculated as the percentage recovery of the analyte in non-spiked serum. The measurement range was defined as the range between LLOQ and ULOQ, which were determined from 10 independent runs with the standard peptide. Measurements below LLOQ or above ULOQ were assigned the value of LLOQ/ULOQ, respectively. IC50 (half-maximal inhibition concentration) was determined from the standard curve. The analyte stability was examined through temperature tests and repeated freeze–thaw cycles of serum samples. The temperature tests included different time point and temperatures where CILP-M levels were measured in three human serum samples after 0-, 2-, 4-, 24-, and 48-h incubation at either 4 °C or 20 °C. The recovery was estimated with 0 h sample as a reference. Furthermore, the effect of four repeated freeze/thaw cycles of three serum samples was assessed where freeze/thaw recovery was calculated with the zero cycle samples as a reference. Each sample was run in double determination.

### In vitro cleavage and Western blot

Articular cartilage biopsies from OA patients who underwent knee replacement surgery were obtained from Gentofte Hospital Denmark. A broad panel of enzymes, known to cleave human articular cartilage was tested^4^. The enzymes included MMP-1, MMP-2, MMP-3, MMP-8, MMP-9, MMP-10, MMP-12, MMP-13, ADAMTS4 and ADAMTS5. The cartilage cleavage was performed as follows; pulverized cartilage samples (30 mg) were incubated with 4 μg of the individual enzyme with 250 μL digestion buffer as previously described^4^. The digestion was carried out for 72 h in replicates. The reaction was stopped by adding 5 mM EDTA. Cleaved products were measured in the CILP-M ELISA and run on Western blot. For the Western blot, the digested samples (1 µg of total protein), and the ladder SeeBlue Plus2 (Thermo Fisher Scientific, Waltham, MA, USA) were electrophoresed on a NuPAGE 4–12% Bis–Tris gel (Invitrogen, Carlsbad, CA, US) under reducing conditions using NuPAGE® MES SDS running buffer (Invitrogen, Carlsbad, CA, US). By using an iBlot® Dry blotting system (Life Technologies, Carlsbad, CA, US), the proteins from the polyacrylamide gel were transferred onto an iBlot® nitrocellulose membrane (Life Technologies, Bengaluru, India). Subsequently, the membrane was blocked for 1h with 5% skim milk (Sigma–Aldrich, St. Louis, MO, USA) in TBST (Tris-Buffered Saline (TBS) with 0.1%. Tween-20). The membrane was incubated overnight at 4 °C with CILP-M mAb. Next, the membrane was washed in TBST three times 10 min and incubated with the secondary peroxidase conjugated antibody (1:5000, Jackson immunoresearch, West Grove, PA, US) for 1 h. The membrane was washed in TBST and incubated for 5 min with SuperSignal west femto maximum sensitivity substrate (Thermo Fisher Scientific, Waltham, MA, USA). The bands were visualized through C-DiGit™ Blot Scanner (LI-COR Biosciences, Lincoln, NE, USA). ImageLab software version 6.1 (Bio-Rad) was used for image acquisition.

### Clinical evaluation of CILP-M

The clinical utility of CILP-M was evaluated in serum samples from a discovery and a validation cohort. The discovery cohort was acquired from the commercial vendor Proteogenex (Culver City, CA). The discovery cohort included a group of healthy donors (n = 13), patients diagnosed with RA (n = 18), patients diagnosed with ankylosing spondylitis (n = 14), and patients diagnosed with osteoarthritis (n = 8). The validation cohort was collected at University of Alberta, Canada, and included serum samples from patients diagnosed with RA (n = 23), patients diagnosed with AS (n = 146), and results were compared with age-matched healthy donors from the commercial vendor BioIVT (Westerbury, NY, USA) (n = 105). In the validation cohort, BASDAI and mSASSS were recorded for each AS patient. The AS samples were assayed prior to the start of treatment with a biologic. 97 patients with AS were treated with anti-TNF-α treatment. However, only 55 of those patients had samples available for CILP-M measurements before and after treatment. To assess the efficacy of anti-TNF-α treatment, patients were categorized as responders and non-responders based on a 50% reduction of the BASDAI score. To evaluate disease progression, patients were categorized as progressors and non-progressors based on whether they showed an increase in the mSASSS score (change of > 0) at the 2-year follow-up. Serum samples were collected and stored at -80 °C until ready for use.

### Ethical statement

All animals were treated according to the guidelines for animal welfare. MAb production in mice was approved by the Danish National Authority (The Animal Experiments Inspectorate) under approval number 2013-15-2934-00956. The collection and retrieval of the human cartilage complied with international ethical guidelines for handling human sample and patient information. All participants signed an informed consent, and the study was approved by the local ethical committee (University of Alberta Health Ethics Review Board) under approval number Pro00000856. Samples from both cohorts were collected after informed consent and approval by the local Ethical Committee and in compliance with the Helsinki Declaration of 1975. The study is reported in accordance with ARRIVE guidelines.

### Statistical analysis

For all statistical analysis performed, a p-value below 0.05 was considered significant. Baseline characteristics are described as number (frequency) and percentage for categorical variables, and as mean (± SD) for continuous variables. Kruskal–Wallis rank test was used to examine baseline differences between groups of participants. One-way ANCOVA analyses were performed in both cohorts (discovery and validation) to measure differences in CILP-M at baseline. Additionally, AUROC analyses were used to investigate the discrimination accuracy of CILP-M between patients and healthy controls at baseline in both studies. These analyses were adjusted for age and sex in both cohorts. Spearman’s correlations were performed with CILP-M and clinical scores (CRP, ESR, disease activity score 28 (DAS28) or BASDAI for RA or AS patients, respectively) at baseline in the validation study. A paired t-test was used to investigate differences in CILP-M levels before and after 3 months of TNF inhibitor (Etanercept, Adalimumab, Infliximab, or Golimumab) in patients with AS in the validation study (patients were biologically naïve prior to treatment).

A linear mixed model was used to evaluate if CILP-M could discriminate response to TNF inhibitor based on 50% reduction of BASDAI, including response and visit as fixed effects and patient specific intercepts as random effect. A t-test was used to explore differences in CILP-M levels between mSASSS progressors and non-progressors.

Statistical analysis and graphs were performed using GraphPad Prism version 9 (GraphPad Software, Inc., La Jolla, CA) and R studio version 4.2.1 (R Foundation for Statistical Computing, Vienna, Austria. URL https://www.R-project.org).

### Supplementary Information

Supplementary Figure S1.

## Data Availability

All data generated or analysed during this study are included in this published article and are available from the corresponding author on reasonable request.
