# Supplementary material for: A novel biomarker of MMP-cleaved cartilage intermediate layer protein-1 is elevated in patients with rheumatoid arthritis, ankylosing spondylitis and osteoarthritis
Source: Sci Rep. 2023 Dec 7;13:21717. doi: 10.1038/s41598-023-48787-x (PMC10709337; doi:10.1038/s41598-023-48787-x)
Supplement: Supplementary file 1 — Supplementary Figure S1. [file 41598_2023_48787_MOESM1_ESM.pdf]

Supplementary Information

A novel biomarker of MMP-cleaved cartilage intermediate layer protein-1 is elevated in patients with rheumatoid arthritis, ankylosing spondylitis and osteoarthritis

Helena Port, Cecilie Møller Hausgaard, Yi He, Walter P. Maksymowych, Stephanie Wichuk, Dovile Sinkeviciute, Anne-Christine Bay-Jensen, Signe Holm Nielsen

Contents

Supplementary Figure S1. Uncropped version of in vitro cleavage of human articular cartilage by enzymes Western blot.

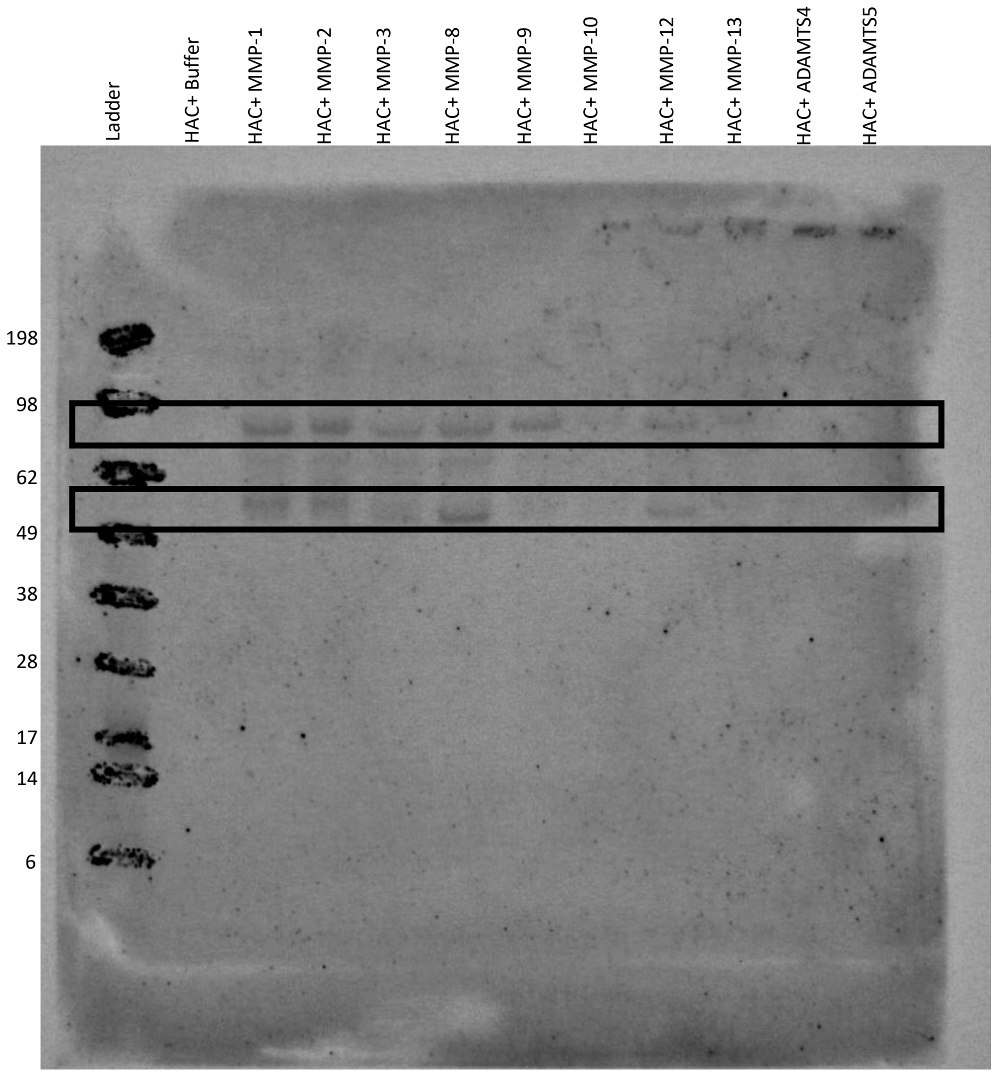

Supplementary Figure 1. Uncropped version of in vitro cleavage of human articular cartilage by enzymes Western blot. Protein expression of CILP-M in human articular cartilage cleaved by MMP1, MMP2, MMP3, MMP8, MMP9, MMP10, MMP12, MMP13, ADAMTS4 and ADAMTS5.
